# Cost-Effectiveness Analysis of COVID-19 Inactivated Vaccines in Reducing the Economic Burden of Ischaemic Stroke after SARS-CoV-2 Infection

**DOI:** 10.3390/vaccines11050957

**Published:** 2023-05-07

**Authors:** Min Du, Chenyuan Qin, Min Liu, Jue Liu

**Affiliations:** 1 Department of Epidemiology and Biostatistics, School of Public Health, Peking University, No. 38, Xueyuan Road, Haidian District, Beijing 100191, China; 2 Institute for Global Health and Development, Peking University, No. 5, Yiheyuan Road, Haidian District, Beijing 100871, China; 3 Department of Global Health and Population, Harvard TH Chan School of Public Health, 677 Huntington Avenue Boston, Boston, MA 02115, USA

**Keywords:** vaccine, ischaemic stroke, COVID-19, cost-effectiveness

## Abstract

Due to significant economic burden and disability from ischaemic stroke and the relationship between ischaemic stroke and SARS-CoV-2 infection, we aimed to explore the cost-effectiveness of the two-dose inactivated COVID-19 vaccination program in reducing the economic burden of ischaemic stroke after SARS-CoV-2 infection. We constructed a decision-analytic Markov model to compare the two-dose inactivated COVID-19 vaccination strategy to the no vaccination strategy using cohort simulation. We calculated incremental cost-effectiveness ratios (ICERs) to evaluate the cost-effectiveness and used number of the ischaemic stroke cases after SARS-CoV-2 infection and quality-adjusted life-years (QALYs) to assess effects. Both one-way deterministic sensitivity analysis and probabilistic sensitivity analysis were performed to assess the robustness of the results. We found that the two-dose inactivated vaccination strategy reduced ischaemic stroke cases after SARS-CoV-2 infection by 80.89% (127/157) with a USD 1.09 million as vaccination program cost, saved USD 3675.69 million as direct health care costs and gained 26.56 million QALYs compared with no vaccination strategy among 100,000 COVID-19 patients (ICER < 0 per QALY gained). ICERs remained robust in sensitivity analysis. The proportion of older patients and the proportion of two-dose inactivated vaccination among older people were the critical factors that affected ICER. This study suggests the importance of COVID-19 vaccination is not only in preventing the spread of infectious diseases, but also in considering its long-term value in reducing the economic burden of non-communicable diseases such as ischaemic stroke after SARS-CoV-2 infection.

## 1. Introduction

Coronavirus disease 2019 (COVID-19) has wreaked havoc around the world for more than three years, which placed huge disease burden and economic loss globally. As of 9 February 2023, more than 755 million people had their health harmed directly as a result of being infected with COVID-19. Apart from the acute symptoms in the first few weeks after infection, up to 70% COVID-19 survivors may still suffer from at least one of the complex post-COVID-19 conditions [1]. The World Health Organization (WHO) defines post COVID-19 condition, also known as long COVID or COVID-19 sequelae, as occurring in individuals with a probable or confirmed history of COVID-19, usually 3 months after the onset of COVID-19 symptoms, where the symptoms persist for at least 2 months and cannot be explained by another diagnosis [2].

Severe acute respiratory syndrome coronavirus 2 (SARS-CoV-2) is highly prothrombotic and may cause microvascular and macrovascular thrombosis in multiple organs [3]. Given the widespread vasculitis described in COVID-19, low-grade endotheliatis may persist during the recovery period, which continues to pose the threat of delayed thrombotic events [4,5]. Persistent coagulation abnormalities and thrombosis were common in long COVID [6,7]. Ischaemic stroke is one of the significant long-term neurological outcomes after SARS-CoV-2 infection [8,9,10,11]. Beghi et al. systematically reviewed acute and post-acute neurological manifestations of hospitalized COVID-19 patients, of which stroke became the most common neurological disease, where ischaemic stroke predominated [11]. A two-year retrospective cohort study enrolled patients with COVID-19 (*n* = 128,437) and matched patients with other respiratory infections (*n* = 128,437) from eight countries [10]. In terms of 6-month hazard ratio, patients with a history of COVID-19 had a significantly increased risk of first ischaemic stroke diagnosis, compared with controls (hazard ratio [HR] = 1.11; 95%CI 1.06–1.17). Specifically, the risks were 1.89 (1.15–3.09) in children (<18 years), 1.12 (1.05–1.21) in adults (18–64 years) and 1.11 (1.04–1.18) in the elderly (≥65 years) [10]. Based on the national healthcare databases of the United States of America (USA) Department of Veterans Affairs, a cohort with 154,068 individuals with COVID-19, and 5,638,795 contemporary controls were built to estimate risks and burdens of incident neurologic disorders at 12 months following acute SARS-CoV-2 infection [12]. People who survived the first 30 days of COVID-19 exhibited increased risk of ischaemic stroke (hazard ratio [HR] = 1.50; 95% CI 1.41–1.61) [12]. Furthermore, the risks of ischaemic stroke at 12 months for people who were non-hospitalized, hospitalized or admitted to intensive care were 1.27 (1.19–1.36), 3.18 (2.76–3.66) and 4.30 (3.38–5.48), respectively [12]. As the severity of COVID-19 increased, the risk of ischaemic stroke also increased in different subgroups [13].

Vaccination is considered to be the most effective and economical strategy to prevent and control epidemics of infectious diseases [14,15,16,17,18]. Two-dose inactivated vaccines are primarily used in China to protect people from infection and severe disease, with CoronaVac (Sinovac, Beijing, China) and COVILO (Sinopharm, Beijing, China) predominating. Vaccines are effective against COVID-19 and its progression to severe disease [15,19,20]. COVID-19 vaccines exhibited a high probability of reducing healthcare costs and increasing quality-adjusted life-years (QALYs) compared to doing nothing [21]. Except for the protection against acute symptoms directly caused by COVID-19, Ziyad et al. found that people who have been vaccinated exhibited lower risk of COVID-19 sequelae than those who were unvaccinated [20,22]. Growing studies have verified that COVID-19 vaccination is associated with better prognosis in patients with ischaemic stroke [20,22,23]. Kim et al. explored the association between COVID-19 vaccination and ischaemic stroke in a retrospective cohort of 592,719 Korean adults, and the adjusted risk of ischaemic stroke was significantly lower among fully vaccinated patients (aHR = 0.40, 95% CI 0.26–0.63) [20]. Two-dose inactivated COVID-19 vaccines may indirectly reduce the incidence of ischaemic stroke by preventing infection and its progression to poor outcomes on the one hand and reducing the incidence of ischaemic stroke directly on the other hand.

As one of the leading causes of death and disability in the world, the direct medical costs and indirect costs of ischaemic stroke cannot be ignored [24,25]. Health economics evaluations in relation to ischaemic stroke have typically focused on a number of treatment regimens, therapeutic agents or diagnostic measures to evaluate their benefits or utility [26,27,28]. Cost-effectiveness analyses of COVID-19 vaccines were mostly limited to the prevention of infection, severe illness, and death [29,30,31]. The decision-analytic Markov model is the most prominent and commonly used model in the field of health economics to select the optimal solution path to maximize net benefits [30,31]. Considering the wide spread of COVID-19 and the necessity for vaccination, linking the vaccination against COVID-19 with long-term symptoms or complications after SARS-CoV-2 infection may become an important direction for future research [32]. Additionally, public COVID-19 fatigue due to the long duration of the pandemic may make subsequent outreach efforts, such as encouraging more people to complete the full initial series of vaccines and booster doses, difficult to conduct [33]. However, few studies have evaluated the cost-effectiveness of COVID-19 vaccines in reducing the substantial economic and health burden associated with ischaemic stroke after SARS-CoV-2 infection.

Therefore, we aimed to explore the cost-effectiveness of the two-dose inactivated COVID-19 vaccine in reducing the economic burden of ischaemic stroke after SARS-CoV-2 infections in China using the decision-analytic Markov model. Our study innovatively proposed a new direction for future research, namely the long-term economic benefits of COVID-19 vaccination in the field of chronic diseases. Additionally, we explored the clinical significance of vaccination from the perspective of long-term health protection through a modelling study and provided an evidence-based basis for vaccination-related education in the burnout stage of epidemic prevention and control, which was helpful to reduce vaccine hesitancy in the public.

## 2. Methods

### 2.1. Theoretical Background

We proposed a health economic hypothesis that needed to be verified, that is, that administering two-dose inactivated COVID-19 vaccines is highly cost-effective in reducing the economic burden of ischaemic stroke after SARS-CoV-2 infection. We implemented this study based on the following theoretical background. SARS-CoV-2 infection. increases the risk of ischaemic stroke, and two-dose inactivated COVID-19 vaccines can reduce the huge economic burden caused by ischaemic stroke after infection through two ways. On the one hand, vaccines can reduce infection and prevent its progression to severe outcomes, thereby indirectly reducing the possibility of ischaemic stroke. The other one is directly reducing the occurrence of post-COVID-19 conditions, including ischaemic stroke.

### 2.2. Study Design

As the most common economic model to select the optimal solution path to maximize net benefits, we adopted the decision-analytic Markov model to accomplish a cohort simulation for the general population, providing innovative evidence on cost-effectiveness analysis of COVID-19 inactivated vaccines in reducing the economic burden of ischaemic stroke after SARS-CoV-2 infection in China. The methodological advantage of the Markov model and its significance for guiding health decision-making is that, compared with traditional cohort studies with a longer follow-up time, which is not conducive to promoting the popularization of vaccination, the Markov model could consider its long-term value of COVID-19 vaccination in reducing the economic burden of non-communicable diseases such as ischaemic stroke after SARS-CoV-2 infection by integrating existing evidence from multi-source cohort studies. The target population of our model was 100,000 COVID-19 cases, and we stratified them by age (<60 years old: 69.8%; ≥60 years old: 30.2%) based on previous reports in China in the early stages of the COVID-19 pandemic [34]. We included two vaccination strategies in total: no vaccination and two-dose inactivated vaccines, and set vaccination rate for the target population based on the proportion of two-dose inactivated vaccination that was reported by the China Center for Disease Control and Prevention on 30 January 2023 [35].

### 2.3. Model Structure

**Figure 1:**
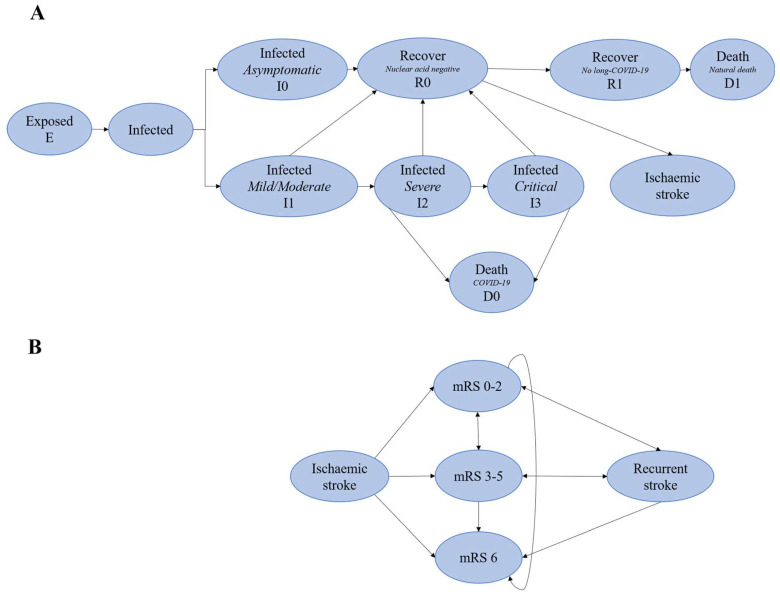
The decision tree model with a long-run Markov state-transition model and ischaemic stroke A. (**A**) Decision tree from natural history of COVID-19, (**B**) Markov state-transition model of ischaemic stroke, patients may have a stable health state, transition to a state of equal or greater disability after recurrent stroke or die. mRS indicates modified Rankin Scale.

A decision tree model with a long-run Markov state-transition model was designed to compare the costs and effects between no vaccination and two-dose inactivated vaccine strategy on ischaemic stroke after SARS-CoV-2 infection. The decision tree was the natural history of COVID-19 infection based on the Susceptible–Exposed–Infected–Recovered (SEIR) structure in Figure 1A [21,36]. The ischaemic stroke incidence rate was obtained from a population-based retrospective study in China [37]. Ischaemic stroke patients would move to one of three possible health states defined by the modified Rankin scale (mRS) including good outcome (mRS 0–2). poor outcome (mRS 3–5) or death (mRS 6) [38,39]. Then, in the long- run Markov state- transition model, patients who survived (mRS 0–2 and mRS 3–5) would enter to evaluate costs and health outcomes in a lifetime horizon. This model used 1-year cycles and would repeat until all patients died theoretically based on the 2020 life expectancy (78 years) in China (17 cycles for old people; the median age of patients was nearly 40 years old among younger patients, so 37 cycles for patients aged <60 years old) [40]. This study assumed that the first ischaemic stroke of patients after SARS-CoV-2 infection progression was the same as the known progression of the disease, as shown in Figure 1B [36,38].

### 2.4. Model Parameters

All parameters of transition probabilities are shown in Supplementary Material Table S1. In the long-run Markov state-transition model, after the first year, ischaemic stroke patients may experience a stable health state, transition to a state of equal or poorer state after recurrent stroke, or die. We assumed that patients in different states (mRS 0–2 and mRS 3–5) had the same risk of recurrence, increased by a factor of 1.03 per life-year. Patients in the mRS 0–2 state who remained alive after stroke recurrence were assumed to be moving between the mRS 0–2 state and mRS 3–5 state, while patients in the mRS 3–5 state who remained alive after stroke recurrence were all assumed to remain in the mRS 3–5 state [36]. Patients in an mRS 0–2 state had the same risk of death as the general population; and patients in mRS 3–5 state have increased mortality compared with patients in an mRS 0–2 state which were adjusted by a factor of 1.68 [39]. We obtained age-specific death rates from the China Population and Employment Statistics Yearbook 2020 [41].

We derived the vaccine effectiveness (VE) of two-dose inactivated vaccination against symptomatic infection from a Randomized Clinical Trial [42]. Parameters of severe diseases, critical diseases and death due to COVID-19 were derived from a real-world study in Chile [43]. There was a lack of VE data of two-dose inactivated vaccination for ischaemic stroke after SARS-CoV-2 infection. Therefore we decided to use the VE data of two-dose mRNA or viral vector vaccination because of their similar VE against severe COVID-19, critical COVID-19 and death due to COVID-19 [44].

In this study, we converted all costs to USD based on the official exchange rates of 2022 (USD 1 US = CNY 6.737) [45]. We measured effectiveness in units of QALYs. According to WHO guide to cost-effectiveness analysis, we set the discount rate for costs and effects as 3% [46,47,48]. Two-dose inactivated vaccines costs included procurement, cold-chain transportation, refrigeration and administration of vaccines [30]. Total costs for ischaemic stroke included the costs for treatment, one-time hospitalization and annual post hospitalization care [39]. Considering deficient Chinese utility scores of different COVID-19 health statuses, we used the utility scores of all health statuses from an Iran study because the Iran population were more similar to the Asian population [49]. The utility of asymptotic patients was referred to the norms for EuroQol Five Dimensions Questionnaire (EQ-5D-5L) of the Chinese general population, given that the general population does not have full health [50]. Utilities for ischaemic stroke patients were assigned to each of the three possible health states for (mRS 0–2, mRS 3–5, mRS 6) [38]. All Parameters of vaccine effectiveness, costs and health utilities are shown in Table S2.

### 2.5. Statistical Analysis

The number of ischaemic stroke cases and QALYs were used to assess effects among 100,000 COVID-19 patients. The model was also purposed to measure the incremental cost-effectiveness ratio (ICER) between the two-dose inactivated vaccination strategy and no vaccination strategy. We determined whether the vaccination strategy was cost-effective by comparing it with the willingness-to-pay (WTP) threshold of the gross domestic product (GDP) per capita of China in 2022 (USD 12,720.49). If the ICER < 0, it considered vaccination strategy as the dominated strategy which can save cost and achieve health outcomes simultaneously. When there were dominant strategies, net monetary benefit (NMB) was evaluated instead. A half-cycle correction was applied to estimate the costs and effectiveness.

Both one-way deterministic sensitivity analysis and probabilistic sensitivity analysis (PSA) were performed to assess the robustness of the results. The one-way sensitivity analysis set plausible ranges of parameters to evaluate the impact of the parameter changes on the ICER. The overall combined uncertainty of all the model parameters were characterized by using Monte Carlo simulations (N = 1000 iterations) in PSA. A cost-effectiveness acceptability curve presented the result of PSA and indicated the probability of cost-effectiveness through a variation of WTP. This study is a Markov model study, and no additional human subject studies were involved. All parameters used in the study were obtained from published literature. Given these reasons, it was exempt from Institutional Review Board (IRB)/ethical approval.

## 3. Results

### 3.1. Costs and Effects of the Two Immunization Strategies

As shown in Table 1, for per 100,000 COVID-19 patients, the total direct cost in the two-dose inactivated vaccination strategy was lower than the no vaccination strategy (USD 245.32 million vs. USD 3919.93 million, respectively). Two-dose inactivated vaccination strategy should pay USD 1.09 million, while saving USD 3675.69 million as direct health care costs. Under the no vaccination strategy, of 100,000 COVID-19 patients, 157 had ischaemic stroke, but under the two-dose inactivated vaccination strategy, only 30 COVID-19 patients developed ischaemic stroke, which decreased by 80.89% (127/157). Meanwhile, two-dose inactivated vaccination strategy gained 26.56 million QALYs, which increased by 403%, compared with no vaccination strategy (5.28 million QALYs).

### 3.2. Cost-Effectiveness Analysis

Compared with no vaccination strategy, two-dose inactivated COVID-19 vaccination strategy generated more QALYs (30.97 vs. 30.87) with lower costs (1165.17 vs. 3279.67). In particular, compared with no vaccination strategy, two-dose inactivated vaccine strategy increased 0.10 QALYs and saved USD 2114.50, with ICER of −20,446.85, respectively (Table 2).

### 3.3. Sensitive Analysis

**Figure 2:**
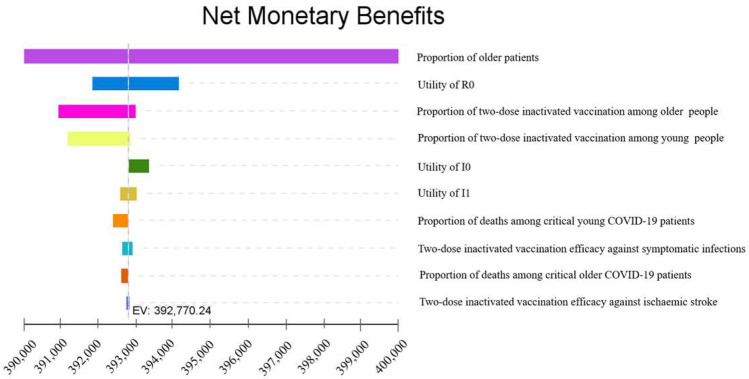
One-way sensitivity analyses of net monetary benefit for the model on willingness to pay. The bars are colored by parameter range. R0: Recovered COVID-19 patients with nuclear acid negative; I0: asymptomatic COVID-19 patients; I1: Mild/Moderate COVID-19 patients; COVID-19: coronavirus disease 2019.

Sensitivity analyses of NMB showed that the proportion of older patients, the utility of R0 and proportion of two-dose inactivated vaccination among older people ranked in the top three that influenced the model most (Figure 2).

**Figure 3:**
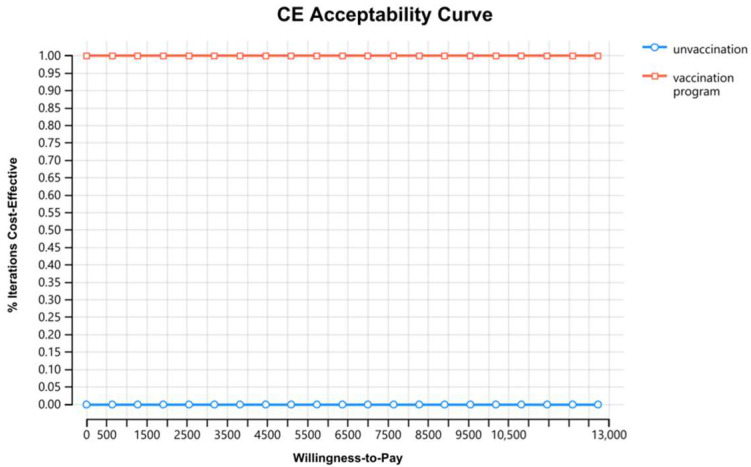
Cost-effectiveness acceptability curve.

PSA presented that, compared with no vaccination strategy, the two-dose vaccination strategy was cost-saving, which could increase more QALYs at the lowest price, and the probability for the two-dose vaccination strategy using the inactivated vaccine being cost-effective was 100% (Figure 3).

## 4. Discussion

To our knowledge, this study innovatively proposed a new direction for future research, namely the long-term economic benefits of COVID-19 vaccination in the field of chronic diseases. Through the decision-analytic Markov model, we found that the two-dose inactivated vaccination strategy reduced ischaemic stroke cases after SARS-CoV-2 infection by 80.89% (127/157) with USD 1.09 million as vaccination cost, saved USD 3675.69 million as direct health care costs and gained 26.56 million QALYs among 100,000 COVID-19 patients. Compared with no vaccination, the two-dose strategy increased 0.10 QALYs and saved USD 2114.50, respectively. The proportion of older patients, the utility of R0 and the proportion of vaccination among older people were the top three factors that influenced the model robustness. The probability for the two-dose vaccination strategies using the inactivated vaccine being cost-effective was 100%. This study validates the high cost-effectiveness of COVID-19 vaccination from a health economics perspective, not only for the acute phase of COVID-19 infection, but also for additional disease and economic burden associated with post COVID-19 conditions (e.g., ischaemic stroke).

Stroke is the leading cause of death and disability in the world and the absolute number of stroke cases increased substantially from 1990 [51]. In 2019, there were 12.2 million (95% CI: 11.0–13.6) incident strokes and 62.4% of them were ischaemic strokes. Statistically, China had the largest number of new cases and deaths related to stroke [51,52]. As one of the long-term outcomes among COVID-19 survivors, the overall incidence rate of new ischaemic strokes in a cohort study of 236,379 survivors was 0.76% (0.68–0.85) [53]. Fortunately, Ziyad et al. found that people who have been vaccinated exhibited lower risk of COVID-19 sequelae than those who were unvaccinated [22]. Combined with the existing global and national disease burden of ischaemic stroke, it is imperative to reduce extra strokes caused by COVID-19 through vaccine strategies. Based on our model, the vaccination strategy showed a commanding advantage in reducing the number of new ischaemic stroke cases belonging to post COVID-19 conditions by more than 80% in China. From *Chinese Health Statistical Yearbook 2019*, the total number of hospitalized patients with ischaemic stroke was more than 3.73 million, and the average hospitalization cost was 9410 yuan (RMB) per capita [24]. Obviously, except for the direct health care costs related to disease treatment, indirect costs cannot be ignored, especially the loss of working time and ability of patients and their families. The American Heart Association reported in 2022 that the annual direct and indirect cost of stroke was over USD 52.8 billion [25]. Given the already high disease and economic burden of ischaemic stroke, our study reported that vaccination program could save nearly USD 3674.61 million in total direct costs at the same time, which indicated that we were supposed to take the long view in reducing the extra costs caused by post COVID-19 conditions via adequate vaccination.

The virus characteristics, acute symptoms, transmission routes, susceptible population and natural and social factors affecting the transmission of SARS-CoV-2 were the research focus in the early stage of this pandemic [54,55,56]. Subsequently, the attention given to SARS-CoV-2 vaccines, especially the health economic evaluation of vaccines, has gradually become a hot topic [17,30,56,57,58]. Vaccination is the most cost-effective way to prevent COVID-19. Fu et al. developed a decision-analytic model to systematically review the cost-effectiveness of inactivated vaccines against COVID-19 in China at a societal perspective [59]. This study provides compelling evidence to support the Chinese free COVID-19 vaccination program that the two-dose inactivated vaccine strategy is effective and cost-saving [59]. However, the vast majority of studies have focused only on the acute infection phase and have not addressed the post-COVID-19 conditions [30,57,59]. Obviously, our modelling study innovatively proposed a new direction for future research, namely, the long-term economic benefits of COVID-19 vaccination in the field of chronic diseases. Another modelling study showed that, in the context of an Australian border reopening plan, long COVID cases would be much lower, even with high rates of infection at the time of reopening, if Australia had a vaccination target of at least 70% and low or moderate public health and social measures implemented [60]. It also verifies the high cost-effectiveness of COVID-19 vaccination in the context of the current resumption of globalization to prevent the additional disease and economic burden of post-infection conditions, such as ischaemic stroke [60].

The results of one-way sensitivity analysis suggested that proportion of older patients and proportion of full vaccination among older people were the two most critical factors affecting ICER. Obviously, for ischaemic stroke, advanced age is the most important independent risk factor [61]. People with hypertension, diabetes and dyslipidemia, which are more common in the elderly population, are also prone to ischaemic stroke [61,62,63]. The proportion of older people who develop symptomatic, severe and critical illness after SARS-CoV-2 infection was significantly higher than that of younger people [64,65]. Simultaneously, above-mentioned patients of COVID-19 were more likely to develop ischaemic stroke as well, both new and recurrent, during the long COVID-19 phase [53]. Previous studies have shown that existing vaccines were effective in older people for the wild virus and various variants in preventing infection, symptomatic infection, hospitalization, and ICU admission [66,67,68,69]. Furthermore, to improve the immunity diminished over time and better fight against more infectious variants, receiving booster doses were strongly recommended [14,70,71]. Given the difficulty of stopping COVID-19 in a short period, the global COVID-19 vaccination process should be accelerated, including full vaccination and booster vaccination. Particularly, it is necessary to expand vaccine coverage among the elderly, who are at high risk of contracting COVID-19 and post-COVID-19 conditions.

From a methodological point of view, we developed a decision-analytic Markov model to evaluate the cost-effectiveness of two-dose COVID-19 vaccination to counter ischaemic stroke after SARS-CoV-2 infection. In particular, the construction of model framework is another extension of the application of Markov model in infectious diseases. Secondly, our study first obtained feasible theoretical hypotheses through a large number of literature reviews, and combined COVID-19, vaccines and post-infectious ischaemic stroke through scientific rigorous logic. Markov model was used to verify that administering COVID-19 vaccines is highly cost-effective in reducing the economic burden of ischaemic stroke after SARS-CoV-2 infection. No economic evaluation of two-dose inactivated COVID-19 vaccines and post-infectious ischaemic stroke has been made in China before. Our study innovatively proposed a new direction for future research, namely the long-term economic benefits of COVID-19 vaccination in the field of chronic diseases. We explored the clinical significance of vaccination from the perspective of long-term health protection through a modelling study and provided an evidence-based basis for vaccination-related education in the burnout stage of epidemic prevention and control, which was helpful to reduce vaccine hesitancy in the public.

This study also had some limitations. First, due to unavailability of parameters, we only explored the cost-effectiveness of two-dose inactivated vaccination strategy in reducing the economic burden of ischaemic stroke after SARS-CoV-2 infection in China. Moreover, due to significant economic burden and disability from ischaemic stroke and strong relationship between ischaemic stroke and SARS-CoV-2 infection, we selected ischaemic stroke as our outcome. In the future, more related research on other severe outcomes is needed. Second, as with all modeling studies, the validity of the results of this study depends largely on the validity and reliability of the parameters in the model. A range of parameter values was considered in sensitivity analyses to assess the robustness of the model. Future studies can further focus on the vaccine strategies with different technical routes, doses and countries.

## 5. Conclusions

In conclusion, our findings suggested that the importance of COVID-19 vaccination is not only in preventing infectious diseases spread, but also considering its long-term value in reducing the economic burden of non-communicable diseases such as ischaemic stroke after SARS-CoV-2 infection. COVID-19 vaccination strategy presented an exceptional potential value to the healthcare system and economy and showed an absolute advantage in reducing total direct cost of ischaemic stroke after infection. To enrich the public health education on the benefit of COVID-19 vaccine and its effectiveness, not only in preventing COVID-19 severe disease and death, but also in reducing stroke incidence due to SARS-CoV-2 infection, it is important to reduce vaccine hesitancy and promote COVID-19 vaccination, especially for elderly people with higher risk of stroke.

## Figures and Tables

**Table 1:** Costs and effects of two-dose inactivated vaccination strategy compared with no vaccination strategy among 100,000 population.

Items	No Vaccination Strategy	Two-Dose Inactivated Vaccination Strategy	Incremental
Cost (2022 USD, million)			
Vaccination cost	0	1.09	−1.09
Direct health care cost	3919.93	244.24	−3675.69
Total direct cost	3919.93	245.32	−3674.61
Effects			
The ischaemic stroke cases after SARS-CoV-2 infection	157	30	−127
Quality-adjusted life-years (million)	5.28	26.56	−21.28

Notes: USD = United States dollar.

**Table 2:** Cost-effectiveness analysis of two-dose inactivated vaccination strategy compared with no vaccination strategy.

Items	No Vaccination Strategy	Two-Dose Inactivated Vaccination Strategy
Cost (USD)	3279.67	1165.17
Effect (QALYs)	30.87	30.97
NMB (USD)	2,232,808.34	2,242,414.92
Incremental Cost (USD)		−2114.5
Incremental Effect (QALYs)	-	−0.1
Incremental cost-effectiveness ratio (ICER, USD/QALY)		−20,446.85

Notes: USD = United States dollar; QALYs = quality-adjusted life-years; ICERs = incremental cost-effectiveness ratios.

## Data Availability

All data from this study are available from the corresponding author upon reasonable request.

## Supplementary Materials

The following supporting information can be downloaded at: https://www.mdpi.com/article/10.3390/vaccines11050957/s1, Table S1: Base-case and plausible ranges of model inputs on transition probabilities; Table S2: Base-case and plausible ranges of model inputs on vaccine effectiveness, costs and health utilities.

## Abbreviations

COVID-19	coronavirus disease 2019
EQ-5D-5L	euroqol five dimensions questionnaireq
GDP	gross domestic product
HR	hazard ratio
ICER	incremental cost-effectiveness ratio
IRB	Institutional Review Board
mRS	modified Rankin scale
NMB	net monetary benefit
QALYs	quality-adjusted life-years
OR	odds ratio
PSA	probabilistic sensitivity analysis
SARS-CoV-2	severe acute respiratory syndrome coronavirus 2
SEIR	susceptible-exposed-infected-recovered
US	United States of America
VE	vaccine effectiveness
WHO	World Health organization
WTP	willingness-to-pay
