# Supplementary material for: Cost-Effectiveness Analysis of COVID-19 Inactivated Vaccines in Reducing the Economic Burden of Ischaemic Stroke after SARS-CoV-2 Infection
Source: Vaccines (Basel). 2023 May 7;11(5):957. doi: 10.3390/vaccines11050957 (PMC10224220; doi:10.3390/vaccines11050957)
Supplement: Supplementary file 1 [file vaccines-11-00957-s001.zip › vaccines-2307755-supplementary.pdf]

Table S1 Base-case and plausible ranges of model inputs on transition probabilities

Model input	Base case	Range	Reference
Proportion of older patients	30.2%	0~100%	[1]
Proportion of two-dose inactivated vaccination	Young people: 90.0%; Older people: 96.6%	0~100%	[2]
Proportion of symptomatic COVID-19	Young people: 66.7%; Older people: 66.2%		[3]
Proportion of severe COVID-19	Young people:8.0%; Older people: 18.7%		[4]
Proportion of critical COVID-19	Young people:2.0%; Older people: 10.6%		[4]
Proportion of critical COVID-19 to death	Young people: 0.63%; Older people: 5.96%		[5]
Ischaemic stroke incidence rate after SARS-Cov-2 infection	Mild/moderate COVID-19: 0.2%; Severe COVID-19: 0.4%; Critical COVID-19: 0.5%		[6]
Proportion of different outcomes of ischaemic stroke at first year	mRS 0-2: 79.6%; mRS 3-5: 13.7%; mRS 6 (death): 6.7%; Recurrent ischaemic stroke: 6.7%	mRS 0-2: 78.0~81.2%; mRS 3-5: 12.4~15.2%; mRS 6 (death): 5.7~7.7%; Recurrent ischaemic stroke: 5.7~7.7%	[7]
Relative risk of stroke recurrence per life-year	1.03	1.02~1.04	[7]
Relative risk of natural death for mRS 3–5 state	1.68	1.49-1.92	[8]
Proportion of patients in recurrent stroke	mRS 0-2: 53.4%; mRS 3-5: 32.4%; mRS 6 (death): 14.2%	mRS 0-2: 47.0~59.6%; mRS 3-5: 26.7~38.6%; mRS 6 (death): 10.2~19.2%;	[8]
Age- specific death rates	See 2020 Census data	-	[9]

mRS, modified Rankin Scale.

Table S2 Base-case and plausible ranges of model inputs on vaccine effectiveness, costs and health utilities

Model input	Base case	Range	Reference
**Vaccine effectiveness**			
Two-dose inactivated vaccination efficacy	Symptomatic COVID-19: 75.0%; Severe COVID-19: 87.5%; Critical COVID-19: 90.3%; Death: 86.3%; Ischaemic stroke: 60.0%	Symptomatic COVID-19: 65.0~83.0%; Severe COVID-19: 86.7~88.2%; Critical COVID-19: 89.1~91.4%; Death: 84.5~87.9%; Critical COVID-19 to ischaemic stroke: 37.0~74.0%	[10-12]
**Cost inputs (2022 US$)**			
Cost of two dose inactivated vaccines	11.8	11.8-14.9	[13]
Cost of COVID-19	Asymptomatic COVID-19: 813.2 Mild/moderate: COVID-19: 915.5; Severe: 8656.4; Critical: 24937.6; Natural death/alive/R0/R1: 0		[14]
Cost of ischaemic stroke	mRS 0–2: 16907.7; mRS 3–5: 18084.5; mRS 6: 15656.5		[7, 8]
**Utility inputs**			
Utility of COVID-19	I0：0.946; I1：0.847<br>I2：0.766; I3：0.629; R0: 0.896; R1/alive：1; natural death/D0/D1：0	I0：0.946~1; I1：0.762~0.932;<br>I2：0.689~0.843; I3：0.566~0.692; R0 0.806~0.986	[15, 16]
Utility of ischaemic stroke	mRS 0–2：0.76；mRS 3–5：0.21；Death (mRS 6)，0	mRS 0–2: 0.69~0.82；mRS 3-5： 0.17~0.26；	[7]
**Discount rate inputs**			
Costs	0.03	0 ~0.06	[17, 18]
Outcomes	0.03	0~0.06	[17, 18]

mRS, modified Rankin Scale.

Reference

1. The epidemiological characteristics of an outbreak of 2019 novel coronavirus diseases (COVID-19) in China. Zhonghua Liu Xing Bing Xue Za Zhi. 2020 Feb 10;41(2):145-51. PMID: 32064853. doi: 10.3760/cma.j.issn.0254-6450.2020.02.003.

2. CDC Weekly. Epidemic situation of COVID-19 in China. URL: https://www.chinacdc.cn/jkzt/crb/zl/szkb_11803/jszl_13141/202302/t20230201_263576.html [accessed 2023-02-01]

3. Ma Q, Liu J, Liu Q, Kang L, Liu R, Jing W, et al. Global Percentage of Asymptomatic SARS-CoV-2 Infections Among the Tested Population and Individuals With Confirmed COVID-19 Diagnosis: A Systematic Review and Meta-analysis. JAMA Netw Open. 2021 Dec 1;4(12):e2137257. PMID: 34905008. doi: 10.1001/jamanetworkopen.2021.37257.

4. Geng M, Ren X, Yu J, Jiang R, Li Z, Feng Z, et al. Clinical characteristics of patients with COVID-19 in different age groups. Disease Surveillance. 2021;36(06):573-80.

5. Baggett TP, Scott JA, Le MH, Shebl FM, Panella C, Losina E, et al. Clinical Outcomes, Costs, and Cost-effectiveness of Strategies for Adults Experiencing Sheltered Homelessness During the COVID-19 Pandemic. JAMA Netw Open. 2020 Dec 1;3(12):e2028195. PMID: 33351082. doi: 10.1001/jamanetworkopen.2020.28195.

6. Wang M, Zhang H, He Y, Qin C, Liu X, Liu M, et al. Association Between Ischemic Stroke and COVID-19 in China: A Population-Based Retrospective Study. Front Med (Lausanne). 2022 Feb 21;8:792487. PMID: 35265632. doi: 10.3389/fmed.2021.792487.

7. Wang Y, Li Z, Zhao X, Wang C, Wang X, Wang D, et al. Effect of a Multifaceted Quality Improvement Intervention on Hospital Personnel Adherence to Performance Measures in Patients With Acute Ischemic Stroke in China: A Randomized Clinical Trial. Jama. 2018 Jul 17;320(3):245-54. PMID: 29959443. doi: 10.1001/jama.2018.8802.

8. Han M, Qin Y, Tong X, Ji L, Zhao S, Liu L, et al. Cost-effective analysis of mechanical thrombectomy alone in the treatment of acute ischaemic stroke: a Markov modelling study. BMJ open. 2022 Apr 6;12(4):e059098. PMID: 35387833. doi: 10.1136/bmjopen-2021-059098.

9. China Population and Employment Statistics Yearbook: China Statistics Press; 2021.

10. Al Kaabi N, Zhang Y, Xia S, Yang Y, Al Qahtani MM, Abdulrazzaq N, et al. Effect of 2 Inactivated SARS-CoV-2 Vaccines on Symptomatic COVID-19 Infection in Adults: A Randomized Clinical Trial. Jama. 2021 Jul 6;326(1):35-45. PMID: 34037666. doi: 10.1001/jama.2021.8565.

11. Jara A, Undurraga EA, González C, Paredes F, Fontecilla T, Jara G, et al. Effectiveness of an Inactivated SARS-CoV-2 Vaccine in Chile. The New England journal of medicine. 2021 Sep 2;385(10):875-84. PMID: 34233097. doi: 10.1056/NEJMoa2107715.

12. Kim YE, Huh K, Park YJ, Peck KR, Jung J. Association Between Vaccination and Acute Myocardial Infarction and Ischemic Stroke After COVID-19 Infection. Jama. 2022 Sep 6;328(9):887-9. PMID: 35867050. doi: 10.1001/jama.2022.12992.

13. Fu Y, Zhao J, Wei X, Han P, Yang L, Ren T, et al. Cost-Effectiveness of COVID-19

Sequential Vaccination Strategies in Inactivated Vaccinated Individuals in China. Vaccines. 2022 Oct 14;10(10). PMID: 36298577. doi: 10.3390/vaccines10101712.

14. Jin H, Wang H, Li X, Zheng W, Ye S, Zhang S, et al. Economic burden of COVID-19, China, January-March, 2020: a cost-of-illness study. Bull World Health Organ. 2021 Feb 1;99(2):112-24. PMID: 33551505. doi: 10.2471/BLT.20.267112.

15. Alinia C, Yaghmaei S, Abdullah FZ, Ahmadi A, Samadi N, Pourteimour S, et al. The health-related quality of life in Iranian patients with COVID-19. BMC infectious diseases. 2021 May 20;21(1):459. PMID: 34016041. doi: 10.1186/s12879-021-06170-z.

16. Xie S, Wu J, Xie F. Population Norms for SF-6Dv2 and EQ-5D-5L in China. Applied health economics and health policy. 2022 Jul;20(4):573-85. PMID: 35132573. doi: 10.1007/s40258-022-00715-2.

17. World Health Organization WH. WHO GUIDE TO COST-EFFECTIVENESS ANALYSIS. URL: https://apps.who.int/iris/bit

stream/handle/10665/42699/9241546018.pdf?sequence=1&isAllowed=y. [accessed 2023-02-07]

18. Jing W, Liu J, Wu Y, Ma Q, Liu M. Cost-effectiveness of couple-based immunization strategy to prevent mother-to-child transmission of hepatitis B virus in China: A decision-analytic Markov model. EClinicalMedicine. 2020 Feb;19:100264. PMID: 32055793. doi: 10.1016/j.eclinm.2020.100264.
